# Lamb performance in hardwood silvopastures, II: animal behavior in summer^1^

**DOI:** 10.1093/tas/txz177

**Published:** 2019-11-26

**Authors:** Gabriel J Pent, Scott P Greiner, John F Munsell, Benjamin F Tracy, John H Fike

**Affiliations:** 1 Shenandoah Valley Agricultural Research and Extension Center, Virginia Polytechnic Institute and State University, Raphine, VA; 2 Department of Animal and Poultry Sciences, Virginia Polytechnic Institute and State University, Blacksburg, VA; 3 Department of Forest Resources and Environmental Conservation, Virginia Polytechnic Institute and State University, Blacksburg, VA; 4 School of Plant and Environmental Sciences, Virginia Polytechnic Institute and State University, Blacksburg, VA

**Keywords:** black walnut, honeylocust, lying down, standing up, time budget

## Abstract

Integrating trees into pastures, a practice known as silvopasture, may benefit livestock in the summertime through the provision of shade. The purpose of this project was to compare the behavioral patterns of sheep grazing in silvopastures and open pastures. Black walnut (*Juglans nigra* L.) and honeylocust (*Gleditisia triacanothose* L.) based silvopasture systems were compared with open pastures in a randomized complete block design with three blocks over two summers. Behavior measures were recorded within a replicate within a week, and these measures were taken sequentially within three experimental periods. Ewe lambs (*n* = 3) within each experimental unit were equipped with a wideband audio-recording device to detect prehension events. Time-lapse cameras documented sheep behavior every 60 s. In the silvopastures, the lambs spent over 90% of daylight hours within shade from trees. Lambs in silvopastures spent more time lying down than animals in the open pastures (*P* ≤ 0.01), while lambs in the open pastures spent more than 2 h longer each day standing (*P* < 0.0001). Lambs in the black walnut silvopastures spent more time grazing (488 ± 14 min · d^−1^) than lambs in the honeylocust silvopastures (438 ± 14 min · d^−1^; *P* = 0.0493) and lambs in the open pastures (417 ± 14 min · d^−1^; *P* = 0.0026). There was no difference in grazing time for lambs in the latter two systems (*P* = 0.5597). Spectral analysis of the imagery revealed that the lambs in the black walnut silvopastures grazed more frequently than the lambs in the other systems for both years. The acoustic analysis, though limited by recorder durability to 47 complete recordings, revealed no difference in total bites taken per day (*P* ≥ 0.7222) or in the morning (*P* ≥ 0.2069), afternoon (*P* ≥ 0.5816), and evening periods (*P* ≥ 0.9337). Silvopastures provide an opportunity to improve lamb comfort in the summer.

## INTRODUCTION

While temperate silvopastures may differ from open pastures in terms of forage yield, composition, or nutritive value, these responses do not necessarily track differences in animal performance between silvopastures and open systems (Peri et al., 2001; Lehmkuhler et al., 2003; Kallenbach et al., 2006; Pent et al., 2019). Research with sheep (*Ovis aries*) and cattle (*Bos taurus*) grazing in hardwood tree-based silvopasture systems suggests animal performance is comparable to that from open pastures, even when forage yield is reduced (Kallenbach et al., 2006; Fannon et al., 2017; Pent et al., 2019). The mechanisms behind these responses have not been clearly defined. Some data suggest that increased crude protein and lower fiber levels in silvopasture forages compensates for lower forage mass in silvopastures (Kallenbach et al., 2006). However, other studies have shown that forages collected from silvopastures have lower soluble carbohydrates (Buergler et al., 2006) and little difference in fiber content and digestibility (Fannon et al., 2017; Pent et al., 2019) compared to forages collected from open pastures.

One cited benefit of silvopasture is the provision of shade to livestock during the summertime (Orefice et al., 2017). Sheep in silvopastures have lower core body temperatures than sheep in open pastures during the afternoon (Pent et al., 2018). Heat load may change activities and intensify stresses experienced by animals in open pastures, thus, increasing time and energy spent in behaviors to stabilize body temperature. Ambient temperatures are lower and less variable in silvopasture systems (Karki and Goodman, 2015); thus, animals may experience more time with conditions suitable for grazing, and dry matter intake (DMI) may, thereby, increase (Mitlohner and Laube, 2003). Altered animal behaviors—such as grazing time, rumination, standing up, and lying down—and consequences to energy expenditure may be an important driver of the similar animal gains observed between open and silvopasture systems. The objective of this study was to use automated technologies to determine differences in lamb behavior within hardwood silvopastures and open pastures. Lambs were expected to be more comfortable in the silvopastures than the open pastures and, thus, were expected to graze more and at different times of the day and spend more time lying down than lambs in the open pastures. Lamb productivity and forage characteristics are available in a corresponding article (Pent et al., 2019).

## MATERIALS AND METHODS

All procedures were approved by the Virginia Tech Institutional Animal Care and Use Committee under protocol number 14–075.

### Research Site

The study was conducted during 12 wk in the summers of 2015 and 2016 at the Whitethorne Agroforestry Demonstration Center, located at Virginia Tech’s Kentland Farm in Blacksburg, VA (37.20 N 80.58 W). Soil series on the site include Berks–Lowell–Rayne complex, Unison and Braddock soils, and Weaver soils, arranged in order of decreasing slopes from 25% to 65%, 15% to 25%, and 0% to 5%, respectively. These soils are generally fine or fine-loamy mixed materials formed along river terraces.

### Pasture and Tree Management

The silvopasture treatments had been established in what was a uniform, cool-season pasture in 1995. Infection levels of tall fescue (*Schedonorus arundinaceus* (Schreb.) Dumont., syn. *Lolium arundinaceum* (Schreb.) Darbysh., formerly *Festuca arundinacea* Schreb.) with an ergot alkaloid-producing endophyte (*Neotyphodium coenophialum* (Morgan-Jones and Gams) Glenn, Bacon, and Hanlin) were greater than 75% for all pastures. The trees were thinned to a final density in 2012, leaving an approximate 12.2- by 12.2-m configuration, with about 36 stems · ha^−1^. Open and silvopasture treatments were replicated three times across the site in a randomized complete block design. The lambs in the open pastures had partial access to shade in the mornings or evening hours when trees within the silvopastures or surrounding woodlots blocked the sun when it was at a low angle in the sky. The total area of each experimental unit (EU) was 0.27 ha · EU^−1^, and each EU was subdivided into eight paddocks for rotational stocking.

Cattle grazed the site once in the spring of each year prior to the grazing study. Following spring grazing with cattle, pastures were clipped with a rotary mower to remove seedheads (15–20 cm). Sheep also grazed the site for 6 wk as part of a second study during fall 2015.

Nitrogen was applied as urea in May 2015 at a rate of 67 kg · ha^−1^. Pastures also were fertilized for a stockpiling study in fall 2015; thus, no fertility was added in spring 2016. Red clover (*Trifolium pratense* L.) seed were broadcast in all EUs at 4 kg · ha^−1^ in the beginning of 2016. Due to undesirable species and associated low productivity in the black walnut (*Juglans nigra* L.) silvopastures, 4.8 kg · ha^−1^ of tall fescue (cv. Kentucky 31) seed and 1.1 kg · ha^−1^ of orchardgrass (*Dactylis glomerata* L. cv. Benchmark+) seed were broadcast over each black walnut silvopasture EU after the summer of 2015, followed by two passes with a drag harrow.

To control yellow crownbeard (*Verbesina occidentalis* (L.) Walter), all pastures were clipped to 13 cm and, 10 d later, all black walnut silvopasture systems and the open system in block 1 were treated with 5 L · ha^−1^ of Weedar 64 2,4-D amine broadleaf herbicide (Nufarm Ltd., Laverton, Australia) using a boom sprayer. Any large spots of stickweed throughout all other EUs were spot-sprayed with the same herbicide mixture using a backpack sprayer. Early in summer 2016, all paddocks were spot sprayed with 23 mL · L^−1^ Weedar 64 2,4-D amine broadleaf herbicide (Nufarm Ltd., Laverton, Australia) using a backpack sprayer, targeting stickweed, Canada thistle (*Cirsium arvense* (L.) Scop.), milk thistle (*Silybum marianum* (L.) Gaertn.), blackberry (*Rubus* spp.), autumn olive (*Elaeagnus umbellate* Thunb.), and any honeylocust (*Gleditisia triacanthose* L.) sprouts in nonhoneylocust silvopasture treatments.

Tree management over the three seasons was largely limited to the winter prior to the 2016 grazing season. Trees in silvopastures were trimmed to maintain clear boles from the ground to the first branch (2.5–5 m height). Stump growth (from trees thinned in 2008 and 2012) was trimmed to 54–60 cm height.

### Sheep

In 2015, Suffolk and Dorset crossbred ewe lambs (*n* = 60) and ram lambs (*n* = 10) were acquired from a farm in Pulaski County, VA. Ram lambs were banded and all sheep were dosed with 8.8 mg · kg^−1^ body weight (BW) of Prohibit Levamisole Drench solution (AgriLabs, St. Joseph, MO) and given a booster vaccination for *Clostridium perfringens* (May 22, 2015). Lambs grazed the adjacent pasture for 8 d before study initiation (May 30, 2015). At 8 wk (July 23, 2015), the lambs’ level of anemia was scored based on the color of their lower eyelids according to the FAMACHA protocol as described by Kaplan et al. (2004). Any lamb with a score of ≥3 received 8.8 mg · kg^−1^ BW of Prohibit Levamisole Drench solution (AgriLabs, St. Joseph, MO). The same deworming protocol was followed at weeks 10 and 12.

In 2016, Dorper and Dorset crossbed ewes lambs (*n* = 49) and wethers (*n* = 21) were acquired from a farm in Scott County, VA. All sheep were dosed with 8.8 mg · kg^−1^ BW of Prohibit Levamisole Drench solution, 0.2 mg · kg^−1^ BW of Cydectin Oral Sheep Drench (Boehringer Ingelheim, Vetmedica, Inc., St. Joseph, MO), and 4.5 mg · kg^−1^ BW of Panacur Sheep Drench (Intervet Inc./Merck Animal Health, Madison, NJ). Lambs received a booster vaccination for *C. perfringens* at the initiation of the study (May 19, 2016). In the second week (June 2, 2016), the lambs’ anemia levels were scored and treatments administered as described, and the same deworming protocol was followed every 2 wk thereafter. Southern States Sheep Mineral with Zinpro (Southern States Cooperative, Inc., Richmond, VA) and water were provided ad libitum to all lambs throughout the duration of the study.

### Stocking Rates and Methods

Each year, lambs were stratified by sex and BW. In the second year, lambs were also stratified by predominant body color (white, black, and tan). Lambs were then randomly assigned to each of the 9 EUs.

Stocking rates for each treatment within years were set based on herbage availability. Forage mass was estimated just prior to study initiation each year. Average lamb weights at the beginning of the study in 2015 and 2016 were 25 and 21 kg, respectively. In the first year, the black walnut silvopastures were stocked with six ewes, while the remaining two treatments were stocked with an additional wether. In the second year, the black walnut silvopastures were stocked with four ewes and one wether, while the remaining two treatments were stocked with five ewes and two wethers.

Sheep in all EUs were moved simultaneously to a fresh paddock once average residual forage heights reached about 7 cm. At the start of each rotation, sheep were allowed access to the half of the paddock where water was available. After about a third of the expected time (about 1–2 d) needed to graze a complete paddock had elapsed, sheep were provided access to the remaining (ungrazed) portion of the paddock. Although this allowed the sheep to back-graze, this was necessary to provide access to water. Back-grazing generally lasted 2–4 d and had little effect on forage production based on visual observation.

### Acoustics

Three sheep in each EU within a block were fitted with a Roland R-05 recorder (Roland Corporation, Los Angeles, CA), which recorded WAV files at a 16-bit resolution and 48-kHz sampling rate, and a Sennheiser ME-2 lavalier microphone (Sennheiser Electronic Corporation, Wedemark, Germany). Recording input volume was set to 80. The recorded was wrapped in bubble wrap packaging and housed in a water-resistant pack strapped to a body harness. The microphone ran through 6-mm plastic tubing to a nylon adjustable halter where it was secured with electrical tape near the mouth of the lamb. Recordings were automatically split after reaching 2 Gb in size. Recorders were usually placed on the animals before 700 h. Recorders were removed from the animals after 2100 h both years, except in the case of rain in 2016. The devices were used to record nine animals for 1 d of week 1. In week 2, the devices were used to record another nine animals in block 2. In week 3, the devices were used to record another nine animals in block 3. This rotation was repeated in weeks 5 and 9.

Recordings were manually analyzed for acoustic integrity, primarily a secure microphone connection. Only sets with completely valid recordings were analyzed further. Valid recordings were reduced to monaural and processed with a high-pass frequency filter (600 Hz, 4800 dB) in Audacity v. 2.0.6 (Audacity Team). A 10-s segment of grazing time was selected from each valid recording and the bites therein were manually counted by listening for a prehension event. Any recording that was too loud or too quiet, based on the determined average threshold of all the recordings, was excluded from the analysis. In SIGNAL and GRASS software (Engineering Design, Berkeley, CA), the selected clip was analyzed for necessary parameters to include all identified bites and exclude all extraneous noise as described in Clapham et al. (2011). The necessary parameters included low- and high-frequency cutoffs, envelope decay time, detection threshold, minimum event gap, minimum pulse length, minimum and maximum event lengths, and pre-event and postevent time extensions. The parameters defining an intake event were slightly different than those established in Clapham et al. (2011). Although the physical characteristics of the acoustic signal of a prehension event remained the same, including the low- and high-frequency cutoffs and the detection threshold, most of the parameters related to the timing of the signals were adjusted to correct for the faster grazing behavior of lambs compared to cows (Table 1). These parameters included envelope decay time, minimum event gap, minimum and maximum event lengths, and pre-event and postevent time extensions. Other timing parameters not adjusted include the minimum pulse length.

**Table 1.** Parameters for defining an intake event in GRASS/SIGNAL software

Parameter	Measurement
Low frequency cutoff, kHz	17
High-frequency cutoff, kHz	None
Envelope decay time, ms	50
Detection threshold, V	0.013
Minimum event gap, ms	40
Minimum pulse length, ms	1
Minimum event length, ms	50
Maximum event length, ms	200
Pre-event time extension, ms	10
Postevent time extension, ms	10

That same segment from which bites had been manually counted was then analyzed automatically with those defined parameters using SIGNAL/GRASS detection software and the results were compared to the manual bite count in Microsoft Excel v. 2013 by a regression of total bite count within the recording as determined by automatic detection compared to manual detection (Microsoft Corporation, Redmond, WA). Finally, prehension events in the entire recordings were detected with SIGNAL/GRASS software. The output recorded each bite event with a start and stop time stamp, along with the voltage of the signal. Data included in the analysis were collected between 700 and 2100 h.

### Imagery

Time-lapse imagery was collected during the same time as the acoustic recordings. Moultrie D-500 trail cameras (EBSCO Industries, Inc., Birmingham, AL) were set to visually encompass the entire paddock containing the lambs with recorders, capturing images every 60 s. Prior to sampling, the sheep of interest were marked with pink, orange, or blue fluorescent spray paint to distinguish sheep within the same EU.

The photos were processed sequentially by manually recording the behavior (standing up, lying down, and grazing) and shade utilization (in the shade, in direct sunlight, and overcast or low sun angle) of each marked sheep by minute. Data included in the analysis were collected between 830 and 2030 h in 2015, while data included in 2016 were collected between 715 and 2045 h. Total time in each behavior was calculated by the summation of total minutes engaged in a given behavior. Shade use was calculated by the summation of total minutes in shade and the minutes of time during which the sky was overcast.

### Statistical Analysis

For the analysis of the acoustic data, a mixed analysis of variance (ANOVA) of daily bite count and bite count in morning, afternoon, and evening periods between treatments was analyzed with PROC MIXED in SAS Studio, v. 3.5 (SAS Inst., Cary, NC). Morning was defined as 700 to 1100 h, afternoon was defined as 1101 to 1600 h, and evening was defined as 1601 to 2100 h. Experimental design was treated as a randomized complete block design with three replications. Least squares means (LS means) and Tukey’s adjusted differences were calculated for each year and treatment combination. Differences were considered significant when *P* ≤ 0.05.

For the analysis of the time-lapse imagery data, a mixed ANOVA of time spent lying down, grazing, standing up, and time in the shade between treatments was analyzed with PROC MIXED in SAS Studio, v. 3.5 (SAS Inst., Cary, NC). Experimental design was treated as a randomized complete block design with three replications and a repeated measures analysis of variance by month. Year was included as a random effect. LS means and Tukey’s adjusted differences were calculated for each year and treatment combination. Differences were considered significant when *P* ≤ 0.05.

Spectral analysis of grazing behavior determined from the imagery was completed to determine the grazing cycles of the lambs in the systems each year as described in Fuller (1976) and Diggle (1990). In spectral analysis, the total variation in a time series is partitioned into sums of squares at each Fourier frequency or *ω =* 2*π*/*n*, where *n* is the total number of observations in the series. At each Fourier frequency, a sum of squares, or ordinate, is calculated using the equation:

$$\begin{array}{l} I\left(\omega \right)=n^{-1}\left[\left\{\underset{t=1}{\overset{n}{\sum }}\;y_{t}cos(\omega t)\right\}+\left\{\underset{t=1}{\overset{n}{\sum }}\;y_{t}sin(\omega t)\right\}\right] \end{array}$$

where *I(ω)* is the Fourier ordinate calculated at each frequency (*ω*), *n* is the number of observations in the series, and *t* is the time in minutes. The spectrum for each lamb and grazing day combination was computed using PROC SPECTRA in SAS 9.4 (SAS Inst., Cary, NC). The ordinates of lambs in a single treatment were averaged to create a composite ordinate at each Fourier frequency usin:

$$\begin{array}{l} \overset{\text{\textasciitilde{}}\text{-}}{I}\;\left(\omega_{j}\right)=\frac{1}{r}\underset{k=1}{\overset{r}{\sum }}\;I_{k}(\omega_{j}) \end{array}$$

where *r* is the number of lamb and day combinations sampled for each treatment, and *I*_*k*_*(ω*_*j*_) is the ordinate of the *k*th series at frequency *ω =* 2*π*/*n*. An *F* test was used to identify ordinates that have a significant effect versus ordinates that are merely white noise (Fuller, 1976).

The average spectrum of the two treatments can be compared using the procedure described in Diggle (1990). The ratio of two spectra is computed by:

$$\begin{array}{l} R\left(\omega \right)=\frac{{\overset{\text{\textasciitilde{}}\text{-}}{I}\;}_{1}(\omega )}{{\overset{\text{\textasciitilde{}}\text{-}}{I}\;}_{2}(\omega )} \end{array}$$

with an *F* distribution of 2*r*_1_, 2*r*_2_ df. The ordinates were averaged and the ratios of these ordinates at each Fourier frequency for each treatment combination were calculated in Microsoft Excel v. 2013. The average ordinates were plotted against period along with the Fisher Kappa test critical value. The ratios of these ordinates were plotted against period along with the 5% and 95% critical values for each treatment combination.

## RESULTS

### Acoustics

**Figure 1.**
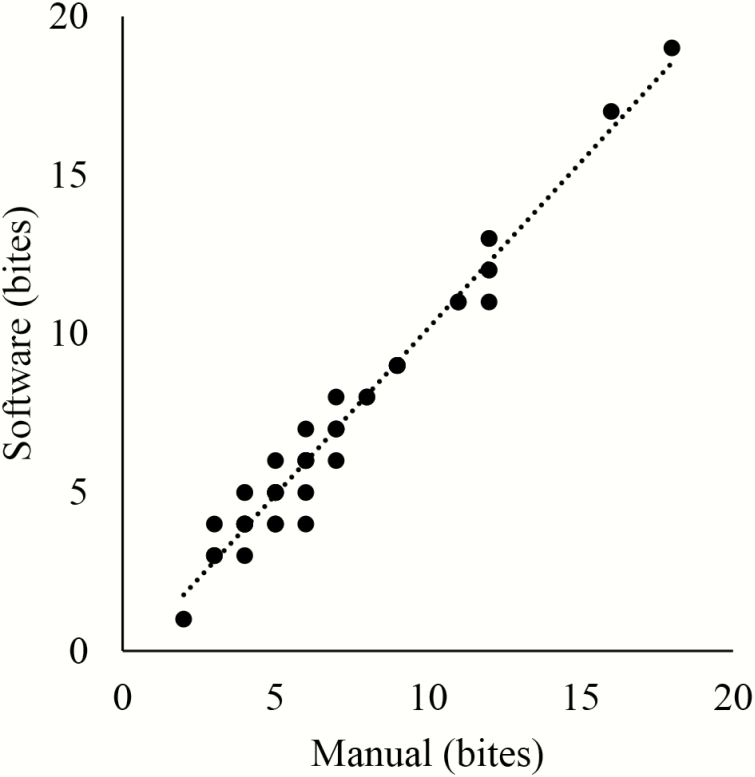
Comparison of manual count of bites to automatic detection of bites with SIGNAL/GRASS software from 47 10-s recordings.

Of the 162 recordings attempted throughout the 2 yr, 47 recordings were valid and complete enough to include in the analysis. The comparison of manually counting prehension events to the automatic detection of prehension events using SIGNAL/GRASS in a short segment from each valid and complete recording is shown in Fig. 1. The *R*^*2*^ of the line was 0.9697. The equation of the line was

$$\begin{array}{l} Automatic\;\;\;event\;\;\;count\;=\;[\;1.05\;\;\times \;\;\left(Manual\;\;\;event\;\;\;count\right)]-0.33 \end{array}$$

**Figure 2.**
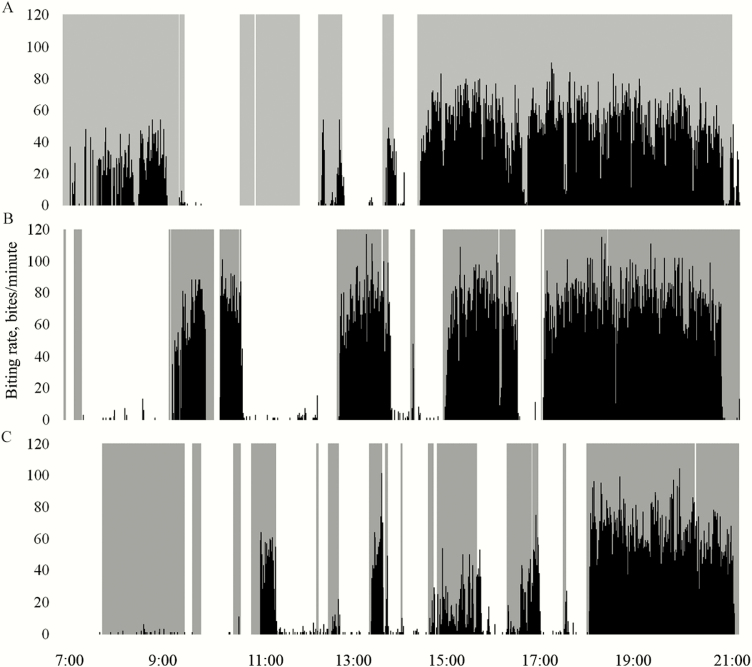
Grazing behavior of three lambs on July 25, 2016. The shaded gray lines indicate time spent grazing as estimated from the time-lapse imagery. The black lines, defined by the right-hand vertical axis, indicate biting rate as detected from SIGNAL/GRASS software. Time of day is defined by the horizontal axis. (A) Ewe No. 4985 in the black walnut silvopastures. (B) Ewe No. 4937 in the honeylocust silvopasture. (C) Ewe No. 4963 in the open pasture.

Of the 47 valid recordings, there were no differences in daily bite count for the lambs in the different systems (Table 2). Figure 2 presents the grazing behavior of three lambs, one from each treatment in the first block, on July 25, 2016. The sheep had been moved to a fresh paddock on July 23, 2016.

**Table 2.** Acoustic analysis of bite counts within periods of day and entire day

	Treatment^1^			Tukey’s adjusted *P*-values^1^		
Year	BW	HL	OP	BW vs. HL	BW vs. OP	HL vs. OP
	Morning ± SE, bites					
2015	1,333 ± 1,112	677 ± 1,492	3,341 ± 1,316	0.9992	0.8494	0.7613
2016	3,659 ± 1,123	1,451 ± 1,047	2,937 ± 786	0.7042	0.9946	0.8625
Total^2^	2,496 ± 790	1,064 ± 911	3,139 ± 766	0.4700	0.8297	0.2069
	Afternoon ± SE, bites					
2015	4,771 ± 1,588	4,849 ± 2,131	3,712 ± 1,879	1.0000	0.9979	0.9985
2016	7,133 ± 1,604	5,136 ± 1,495	7,518 ± 1,123	0.9405	1.0000	0.7962
Total^2^	5,952 ± 1,129	4,992 ± 1,301	5,615 ± 1,095	0.8436	0.9750	0.9289
	Evening ± SE, bites					
2015	9,747 ± 1,965	9,925 ± 2,636	9,018 ± 2,325	1.0000	0.9999	0.9998
2016	12,072 ± 1,984	10,874 ± 1,849	13,267 ± 1,389	0.9976	0.9960	0.9022
Total^2^	10,910 ± 1,396	10,399 ± 1,610	11,142 ± 1,354	0.9689	0.9921	0.9337
	Daytime ± SE, bites					
2015	15,852 ± 4,157	15,451 ± 5,577	16,070 ± 4,919	1.0000	1.0000	1.0000
2016	22,865 ± 4,198	17,460 ± 3,912	23,722 ± 2,939	0.9321	1.0000	0.7933
Total^2^	19,358 ± 2,954	16,455 ± 3,406	19,896 ± 2,865	0.7973	0.9906	0.7222

^1^Treatment: BW = black walnut silvopasture; HL = honeylocust silvopasture; OP = control (open pasture).

^2^Presented by year and by all years combined because of no treatment by year interaction in statistical model.

### Imagery

In 2015, a total of 22, 22, and 26 grazing days were recorded and analyzed with the trail cameras in the black walnut silvopastures, the honeylocust silvopastures, and the open pastures, respectively. In 2016, a total of 27, 24, and 27 grazing days were recorded and analyzed in the black walnut silvopastures, the honeylocust silvopastures, and the open pastures, respectively.

Lambs in the silvopastures actively followed the shade from the trees, spending over 90% of the day within shade boundaries (Table 3). Lambs in the open pastures spent significantly less time within shade than the lambs in the silvopastures. The average number of overcast minutes in 2015 was 167 min · d^−1^, while the average for 2016 was 259 min · d^−1^.

**Table 3.** Time-lapse imagery analysis of behavior and time in shade

	Treatment^1^			Tukey’s adjusted *P*-values^1^		
Year	BW	HL	OP	BW vs. HL	BW vs. OP	HL vs. OP
	Shade use ± SE, minutes					
2015	655 ± 9	681 ± 9	166 ± 5	0.3093	<0.0001	<0.0001
2016	756 ± 5	748 ± 8	216 ± 5	0.9636	<0.0001	<0.0001
Total^2^	705 ± 5	715 ± 6	191 ± 4	0.4653	<0.0001	<0.0001
	Lying ± SE, minutes					
2015	307 ± 24	333 ± 23	162 ± 22	0.9669	0.0010	<0.0001
2016	189 ± 22	254 ± 23	198 ± 22	0.3330	0.9998	0.4863
Total^2^	248 ± 16	294 ± 16	180 ± 16	0.1290	0.0118	<0.0001
	Standing ± SE, minutes					
2015	36 ± 12	43 ± 11	252 ± 10	0.9983	<0.0001	<0.0001
2016	20 ± 9	24 ± 11	80 ± 9	0.9997	0.0009	0.0052
Total^2^	28 ± 8	33 ± 8	166 ± 7	0.8712	<0.0001	<0.0001
	Grazing ± SE, minutes					
2015	376 ± 22	343 ± 21	304 ± 19	0.8930	0.1610	0.7423
2016	601 ± 19	532 ± 21	530 ± 19	0.1617	0.1122	1.0000
Total^3^	488 ± 14	438 ± 15	417 ± 14	0.0493	0.0026	0.5597

^1^Treatment: BW = black walnut silvopasture; HL = honeylocust silvopasture; OP = control (open pasture).

^2^Presented by year and by all years combined despite treatment by year interaction in statistical model.

3Presented by year and by all years combined because of no treatment by year interaction in statistical model

The lambs in the silvopastures spent more time lying down than lambs in the open pastures in 2015 but not 2016. For both years, however, lambs in the open pastures spent more time standing up than lambs in the silvopastures, though the time spent standing was greater in 2015 than in 2016 for lambs in all treatments, particularly the lambs in the open pastures.

Lambs in the black walnut silvopastures spent the most time grazing. Lambs in the honeylocust silvopastures spent as much time grazing as the lambs in the open pastures.

**Figure 3.**
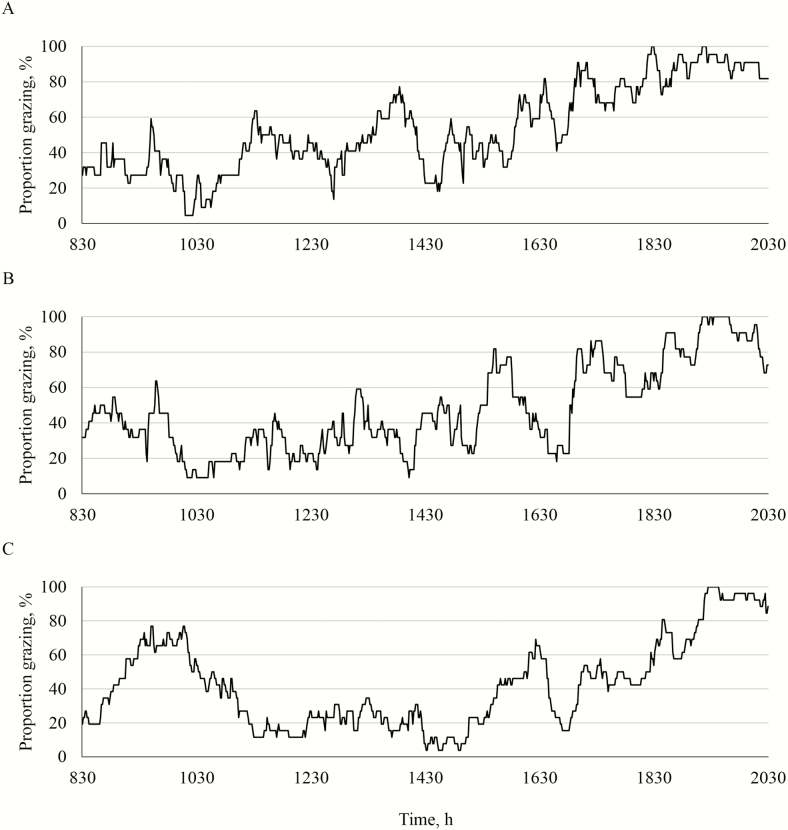
Proportion of total animals grazing each minute in the black walnut silvopastures (A), the honeylocust silvopasture (B), and the open pasture (C) in 2015 from the analysis of the time-lapse imagery.

The proportion of total animals grazing by minute in 2015 is shown in Fig. 3. In 2015, the lambs in silvopastures grazed more evenly than lambs in the open pastures, although the largest grazing bout occurred in the evening for all animals. The lambs in the open pastures appeared to graze less in the middle of the day than the lambs in the silvopastures, particularly than the lambs in the black walnut silvopastures. The evening grazing bout started at a later time in the afternoon for lambs in the open pastures compared to lambs in the silvopastures. The lambs in the open pastures also had a dominant grazing bout in the morning, which was not as evident for lambs in the silvopastures.

**Figure 4.**
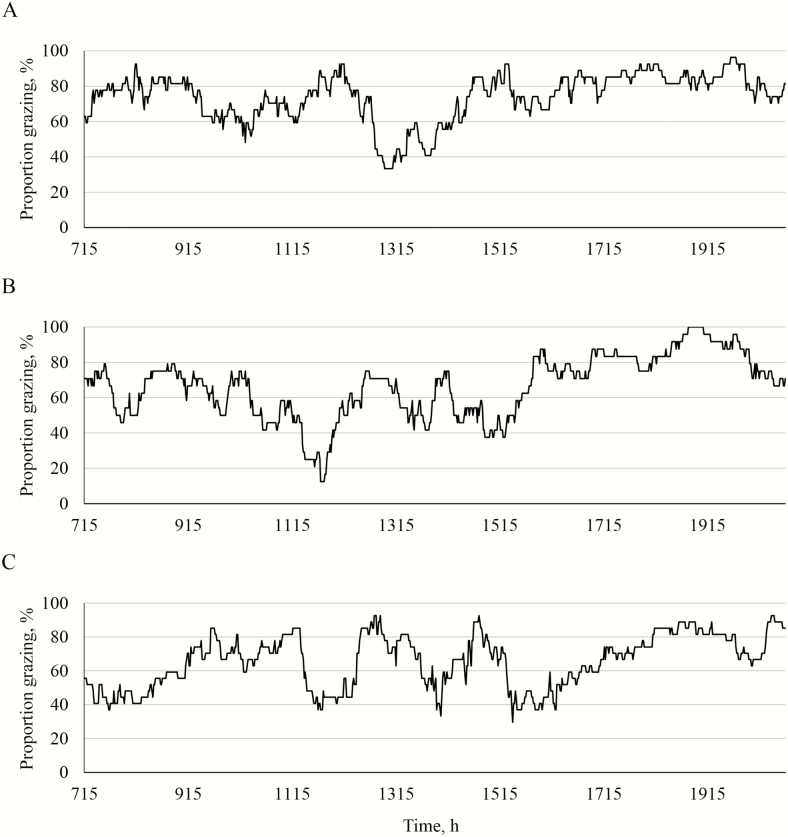
Proportion of total animals grazing each minute in the black walnut silvopastures (A), the honeylocust silvopasture (B), and the open pasture (C) in 2016 from the analysis of the time-lapse imagery.

The proportion of total animals grazing by minute in 2016 is shown in Fig. 4. In 2016, the lambs in the black walnut silvopastures grazed more evenly throughout the day than lambs in the other systems. Lambs in the black walnut silvopastures had four dominant grazing bouts, including one in the morning, one in the early afternoon, one in the late afternoon, and the largest grazing bout in the early evening. Lambs in the honeylocust silvopastures appeared to graze throughout the day, but most of the time spent grazing by these lambs occurred in the final grazing bout. Lambs in the open pastures appeared to have more distinct grazing bouts than the lambs in the other systems, with a grazing bout in the morning, two grazing bouts in the afternoon, and a final evening grazing bout that started later than the final grazing bout for the lambs in the silvopastures.

**Figure 5.**
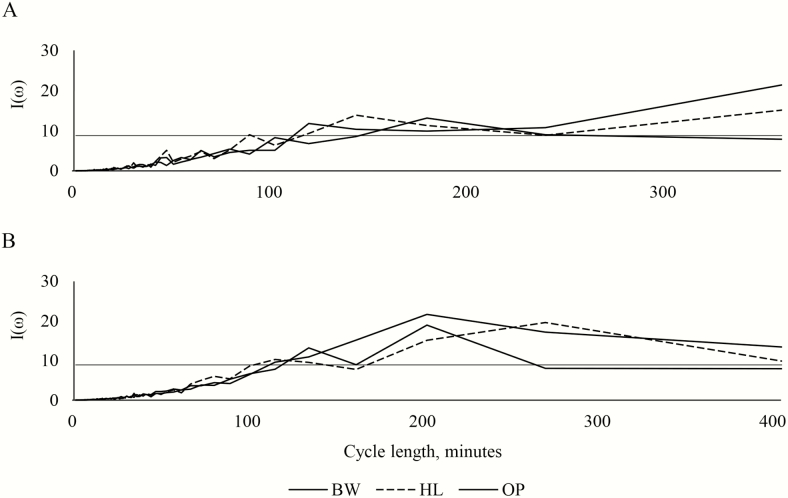
Periodogram ordinates at each Fourier frequency cycle length in 2015 (A) and 2016 (B) from the analysis of the time-lapse imagery. The *F* critical value (*P* < 0.05) is denoted by the horizontal line. Ordinates exceeding this critical value indicate Fourier frequencies that significantly contribute to grazing cyclicity. BW = black walnut silvopasture, HL = honeylocust silvopasture, OP = open pasture.

The composite periodograms of lamb grazing behavior by treatment in 2015 and 2016 with the *F* critical value (8.776 and 8.901, respectively) are plotted in Fig. 5. In 2015, the lambs in the honeylocust silvopastures displayed significant ordinates at frequencies of 90.125, 120.167, 144.2, 180.25, and 240.333. The lambs in the black walnut silvopasture had similar significant ordinates with the exception of the ordinate at frequency 90.125. The lambs in the open pastures had significant ordinates only at the frequencies 180.25 and 240.333. In 2016, the lambs in the open pastures had significant ordinates at frequencies 115.857, 135.167, 162.2, 202.75, and 270.333. The lambs in the black walnut silvopastures were similar but did not display significant ordinates at frequencies 115.857 and 270.333. The lambs in the honeylocust silvopastures were also similar but did not display significant ordinates at frequency 162.2.

**Figure 6.**
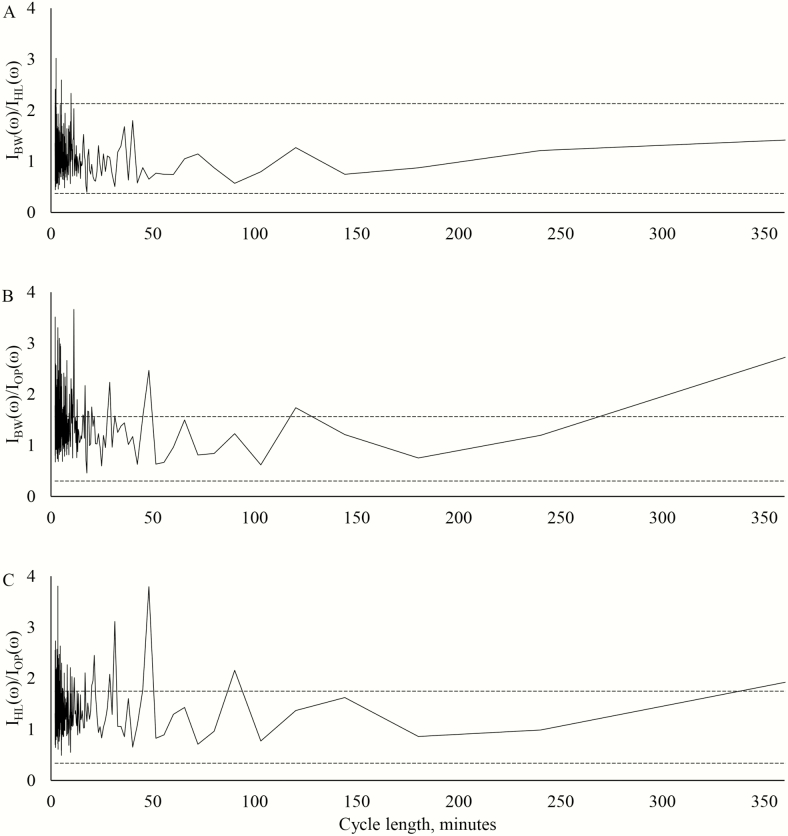
Plots of ordinate ratios at each Fourier frequency cycle length in 2015 from the analysis of time-lapse imagery. The upper and lower *F* critical boundaries (*P* < 0.05) are denoted by the dashed horizontal lines. Ordinate ratios falling outside these boundaries indicate Fourier frequencies where the two spectra are significantly different. (A) Ratio of black walnut silvopasture ordinates to honeylocust silvopasture ordinates. (B) Ratio of black walnut silvopasture ordinates to open pasture ordinates. (C) Ratio of honeylocust silvopasture ordinates to open pasture ordinates.

The ratios of the ordinates for the different treatments in 2015 with the 5% and 95% critical limits are plotted in Fig. 6. Lambs in the black walnut silvopastures grazed more frequently than lambs in either other treatment with significantly larger ordinate ratios at the early frequencies. The ordinate at frequency 120.167 was also significantly larger for the lambs in the black walnut silvopastures compared to the lambs in the open pastures. A similar phenomenon was noticed for the lambs in the honeylocust silvopasture at early frequencies and frequency 90.125.

**Figure 7.**
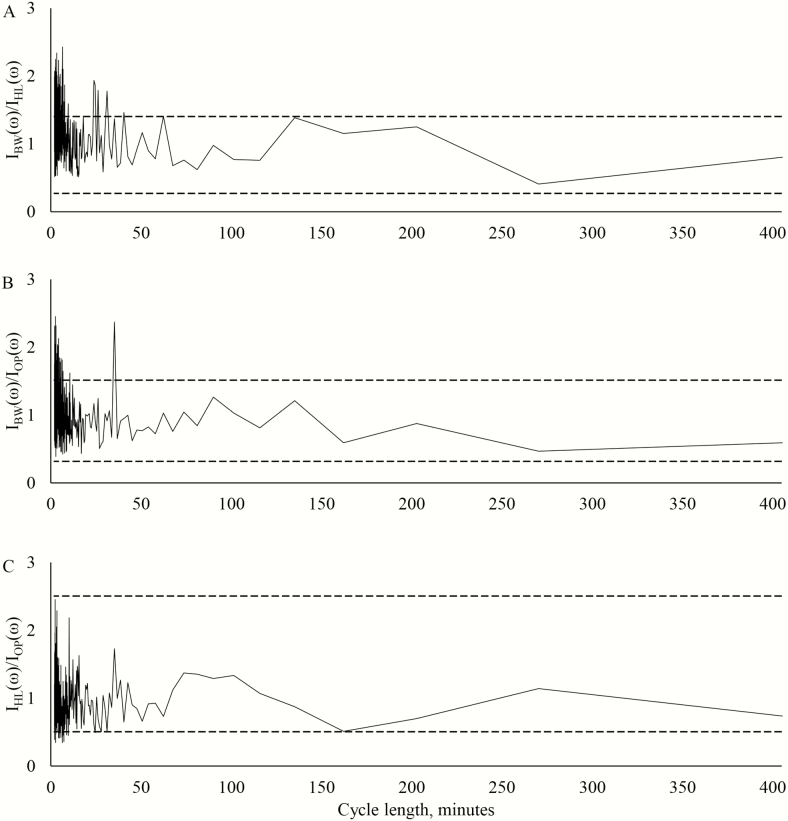
Plots of ordinate ratios at each Fourier frequency cycle length in 2016 from the analysis of time-lapse imagery. The upper and lower *F* critical boundaries (*P* < 0.05) are denoted by the dashed horizontal lines. Ordinate ratios falling outside these boundaries indicate Fourier frequencies where the two spectra are significantly different. (A) Ratio of black walnut silvopasture ordinates to honeylocust silvopasture ordinates. (B) Ratio of black walnut silvopasture ordinates to open pasture ordinates. (C) Ratio of honeylocust silvopasture ordinates to open pasture ordinates.

The ratios of the ordinates for the different treatments in 2016 with 5% and 95% critical limits are plotted in Fig. 7. Lambs in the black walnut silvopastures again grazed more frequently than lambs in either other treatment. During this year, however, the lambs in the open pastures grazed slightly more frequently than the lambs in the honeylocust silvopastures.

## DISCUSSION

### Acoustics

Although the parameters for defining an intake event in the GRASS/SIGNAL software varied from those utilized by Clapham et al. (2011), this was the case both to take into account the more intense biting rate of sheep (Orr et al., 1997) compared to cattle (Erlinger et al., 1990) and because more bite events were accurately detected with these changes. Biting rates were similar in this study to other estimates of biting rates in sheep (Champion et al., 1994; Orr et al., 1997). The correlation between manually comparing bite counts in a recording to the automatic detection of bites in a recording indicates the high level of accuracy that this method is capable of producing. The challenges faced throughout the study were keeping the recorders running and minimizing the variability between recordings, including distance to the noise source and device settings and functions.

There were no differences detected between treatments in total bites taken per period of the day and for the entire day, and this was largely a function of the variability between measurements. Fasting—or time since the last rotation in a rotational stocking system—is a major driver of grazing time in ruminants (Newman et al., 1994), and it seems that this effect precluded our ability to detect differences between treatments in addition to the low number of complete recordings included in the final analysis.

From this limited analysis, however, it appears that the presence of trees does not inhibit grazing. Some studies indicate that dairy cattle, by seeking artificial shade, might suffer reduced intake levels during the day (Kendall et al., 2006). In silvopastures, where shade is evenly distributed throughout the pasture along with adequate forage for intake, grazing time has, in some cases, been found to be greater than in open pastures (Karki and Goodman, 2010).

### Imagery

The lambs in the silvopastures actively followed the shade of the trees as the sun moved throughout the day, spending over 90% of daylight hours within the boundaries of the shade. This study substantiates previous evidence that livestock, including sheep and cattle, prefer shade and will actively seek it in order to minimize heat stress (Roman-Ponce et al., 1977; Morrison, 1983; Bennet et al., 1985; Johnson, 1987; Blackshaw and Blackshaw, 1994). Shade use increased for lambs in all treatments during 2016, although this was primarily driven by an increased number of overcast days. The preference that dairy cattle exhibit for shade increases with rising temperatures and shade availability (Kendall et al., 2006; Tucker et al., 2008; Schütz et al., 2014). This study and others have demonstrated that silvopastures, by taking advantage of the shade preference of livestock and through an even distribution of shade across the landscape, can increase site utilization by livestock while evenly distributing urine and manure (Karki and Goodman, 2010). Preference alone, however, does not indicate greater levels of animal welfare (Broom, 1988).

The lambs in the silvopastures also spent more time lying down than the lambs in the open pastures. Time spent lying down is a traditional metric of animal comfort (Haley et al., 2000; Winckler et al., 2003). In addition, conductive heat loss with the ground is only an effective means of cooling if the ground is cooler than the body of the lamb. As might be expected, soil surface temperatures are generally lower in silvopastures and greater in open pastures (Buergler et al., 2006). As a result, ground within the silvopastures provides a more effective means of conductive heat loss than ground in the open pastures.

Conversely, the lambs in the open pastures spent more time standing up than the lambs in the silvopastures. Standing up is a general response of livestock to heat stress (Cook et al., 2007; Scaglia and Boland, 2014). Greater time spent standing indicates the level of heat stress experienced by the lambs in the open pastures as they sought to increase the effectiveness of convective heat loss through improved airflow (Silanikove, 2000).

Cattle grazing tall fescue infected with an ergot alkaloid-producing endophyte, which increases their susceptibility to heat stress, have been shown to spend less time grazing and lying down and more time standing up compared to cattle grazing nonendophyte-infected tall fescue (Coffey et al., 1992; Seman et al., 1997). This present study compared only wild-type endophyte-infected tall fescue across treatments; it remains to be seen how trees in nontoxic endophyte-infected tall fescue pastures affect animal behavior and performance.

It is interesting to note that while all animals spent less time standing up in 2016 compared to 2015, this discrepancy was particularly evident for the lambs in the open pastures. The lambs in the open pastures also spent more time lying down in 2016, while the lambs in the silvopastures spent less time lying down that same year compared to 2015. This phenomenon occurred despite more moderate Temperature Humidity Index (THI) conditions in 2015 than in 2016 (Pent et al., 2019). The likely reason for this phenomenon was that a hair sheep breed was used in the second year, which is generally more heat tolerant than wool sheep. Wool impairs the effectiveness of sweating as a means of evaporative cooling (Marai et al., 2007). This effect was also evident in the live weight gains of the animals. The difference in average daily gains (ADG) of the lambs in the silvopastures compared with those of the lambs in the open pastures was greater in 2015 than in 2016 (Pent et al., 2019). In a hot environment, animal utilization of shade coupled with more time spent resting and less time spent standing indicates that the animals in the silvopastures were more comfortable (Broom, 1988; Silanikove, 2000).

The lambs in the black walnut silvopastures spent more time grazing, although this is merely a rough estimate of grazing time and was hindered by both obscurity of the field of view within the photos and the challenge of differentiating grazing time from time spent standing. The potential inaccuracies in documenting grazing time from still photos is evident in Fig. 2, where it is evident that, at some points in time, the acoustic analysis and time-lapse imagery analysis do not concur. In addition, grazing time does not necessarily reflect intake, which is dependent, not just on grazing time and grazing intensity but also on bite size. Nevertheless, the greater time spent grazing by lambs in the black walnut silvopastures may have been a function of more comfortable conditions, as has been demonstrated in feedlots where nutrition is not limiting (Mitloehner et al., 2001; Gaughan et al., 2010; Blaine and Nsahlai, 2011), or a lower forage sward, which would require more time spent grazing to compensate for reduced bite size (Allden and McDWhittaker, 1970; Karki and Goodman, 2010). Where forage sward conditions were more similar to those of the open pastures, the lambs in the lighter shade of the honeylocust silvopastures spent the same amount of time grazing as the lambs in the open pastures. When shade is provided apart from feed, as in the case of shade structures provided away from pastures, dairy cattle will spend the majority of the daylight hours in the shade which will negatively affect their daytime grazing behavior (Kendall et al., 2006). Such phenomenon would not be expected in silvopastures, where feed and shade are available in the same locales.

Significantly large ordinates occurred for lambs in all treatments at frequencies of around 2 to 3 hours, indicating the shorter grazing cycles displayed by lambs compared with cattle (Seman et al., 1997). Others have noted, however, that the grazing cycles of sheep followed 8-h cycle lengths (Champion et al., 1994). The data presented in the current study only includes daytime data, which may account for the discrepancy. Eight is a harmonic of a 24-h d. In addition, the present study utilized rotational stocking management, while Champion et al. (1994) utilized continuous stocking management with a relatively even forage sward height.

Lambs in the black walnut silvopastures grazed more frequently than lambs in either of the other treatments in both years. A general response of heat-stressed animals is to reduce intake, and this occurs both indirectly through a reduction in passage rate and directly through an elevation of body temperatures (Silanikove, 1992; Blackshaw and Blackshaw, 1994). The lambs in the black walnut silvopastures also grazed more evenly throughout the day than lambs in the open pastures, which appeared to graze most during the cooler evening hours and generally delayed the final grazing bout relative to the lambs in the silvopastures. Seeking shade and minimizing intake during the hottest times of the day has been understood as an adaptive mechanism for maintaining adequate levels of feed intake during periods of heat stress (Silanikove, 1992). All animals appeared to graze the most during the evening grazing bout, a pattern of activity that has been well established in grazing ruminants (Orr et al., 1997; Gregorini et al., 2006). This pattern has been explained as a mechanism for maximizing energy intake as forage carbohydrate levels increase during the day and decrease during the night. However, the importance of minimizing activity and intake during periods of heat stress may have been underestimated or even ignored in determining the reasons for these diurnal cycles.

Lambs in the honeylocust silvopasture grazed less frequently than lambs in the open pastures in 2016. The switch to a more heat-tolerant sheep breed was a likely reason that the lambs in the open pastures displayed behavior less indicative of heat-stressed animals than in the previous year.

## CONCLUSION

The methods used for determining lamb grazing behavior were well correlated, but the analysis of time-lapse imagery permitted a more complete analysis of daily time budgets than the acoustic analysis. This was due both to the additional information provided by the time-lapse imagery on time spent in the shade, standing, and lying down and the low number of complete audio recordings that were collected. From the acoustic analysis, no differences were found in daily bite counts and bite count by time of day for the lambs. From the analysis of the time-lapse imagery, it was found that the lambs preferred shade and actively sought it throughout the day. Lambs in silvopastures were more comfortable, spending more time lying down and less time standing up than lambs in open pastures. Lambs in black walnut silvopastures, where shade is deeper than the shade available from honeylocust trees, also grazed more frequently and more evenly throughout the midday hours compared to the lambs in the other systems.
